# *Mycobacterium intermedium* Granulomatous Dermatitis from Hot Tub Exposure

**DOI:** 10.3201/eid1205.051281

**Published:** 2006-05

**Authors:** Randall S. Edson, Christine L. Terrell, W. Mark Brutinel, Nancy L. Wengenack

**Affiliations:** * Mayo Clinic, Rochester, Minnesota, USA

**Keywords:** Acid-fast, atypical mycobacterium, cutaneous, dermatitis, granulomatous, hot tub, mycobacteriosis, Mycobacterium intermedium, waterborne, whirlpool, dispatch

## Abstract

Nontuberculous mycobacteria, which are widespread in the environment, frequently cause opportunistic infections in immunocompromised patients. We report the first case of a patient with chronic granulomatous dermatitis caused by a rarely described organism, *Mycobacterium intermedium*. The infection was associated with exposure in a home hot tub.

Nontuberculous mycobacteria are major causes of opportunistic infection in immunocompromised patients. These organisms are widespread throughout the environment, including water and soil (*1**,**2*). We report the first case of a patient with chronic granulomatous dermatitis caused by a rarely described organism, *Mycobacterium intermedium*, which was associated with exposure in a home hot tub.

## The Study

A 55-year-old nonimmunosuppressed man first sought medical attention in March 2000 for an indurated papular rash on his back (Figure, panels A and B). A biopsy showed granulomatous dermatitis, but all cultures and stains, including those for fungi and mycobacteria, were negative. In September 2000, another biopsy showed histologic results identical with those of the previous biopsy. Mycobacterial cultures at this time were positive for *M*. *intermedium*; tissue stains were negative.

**Figure:**
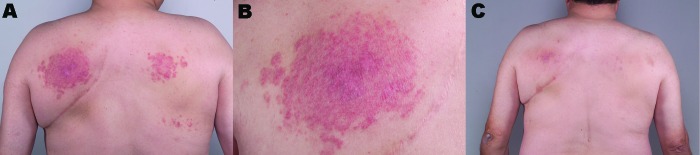
A) Appearance of rash on the patient's back at initial treatment. B) Close-up of the rash shown in panel A. C) Patient's back showing near resolution of rash after discontinuing use of the hot tub.

To confirm the microbiologic result, another biopsy was performed in October 2000, at which time a single auramine-rhodamine–positive staining result was noted in the tissue. Culture at this time was negative for mycobacteria. He was treated with topical corticosteroids with partial improvement. In March 2001, *M*. *intermedium* was recovered from 2 separate biopsy specimens. On the basis of susceptibility data, treatment with isoniazid, ethambutol, and clarithromycin was initiated.

In July 2001, considerable improvement was noted. That same month, the patient received methylprednisolone (1 g intravenously) for 5 days for an ill-defined neurologic condition. At a follow-up visit in June 2002, he reported a 2-week history of diminished vision in the left eye. Ethambutol, isoniazid, and clarithromycin were withdrawn. At that time, his dermatitis was somewhat improved but not entirely resolved. In October 2002, he came to the clinic with new lesions on his back. During this visit, he reported immersion twice a day in a home hot tub, which provided temporary relief for his chronic back pain. Additionally, he reported that when sitting in the tub, his upper back was in contact with several nozzles that delivered water under high pressure. He was advised to refrain from using the hot tub.

Three months later, he had almost complete resolution of the skin lesions with no further medical treatment (Figure, panel C). At that time, a water sample obtained from the patient's hot tub was positive for *M*. *intermedium*.

Skin lesion biopsy specimens were placed in sterile beef nutrient broth and cultured in a mycobacteria growth indicator tube (MGIT, Becton Dickinson, Sparks, MD, USA) supplemented with oleic acid, albumin, dextrose, catalase growth supplement (OADC, Becton Dickinson), and an antimicrobial drug mixture (polymyxin B, amphotericin B, nalidixic acid, trimethoprim, and azlocillin [PANTA, Becton Dickinson]. The MGIT was incubated on the BACTEC MGIT 960 instrument (Becton Dickinson) at 37°C, and growth was shown by an increase in fluorescence after 9 days. A cytospin slide prepared from the broth was positive for acid-fast bacilli by Kinyoun stain. The MGIT broth was subcultured to a Middlebrook 7H10/S7H11 agar biplate and incubated at 37°C in 5% CO_2_ to obtain a pure culture of the organism for subsequent identification.

To obtain a culture of hot tub water, the patient was instructed to fill the hot tub with water and allow it to stand for 2 weeks before sampling. A sample of water was collected from the hot tub in sterile, screw-top tubes. The water was concentrated by centrifugation and treated with N-acetyl-L-cysteine and 1% sodium hydroxide to remove bacteria that might overgrow more slowly growing mycobacteria. The specimen was then injected into an MGIT for incubation at 37°C. The MGIT showed fluorescence (bacterial growth) after 1 day of incubation, and the Kinyoun stain was positive for acid-fast bacilli. The MGIT broth was subcultured to a Middlebrook 7H10/S7H11 biplate and incubated at 37°C in 5% CO_2_ to obtain a pure culture of the acid-fast bacilli for identification.

The skin lesion and hot tub isolates were tested by using nucleic acid hybridization probes (AccuProbe, Gen-Probe Inc, San Diego, CA, USA) to rule out *M*. *tuberculosis* complex, *M*. *avium* complex, and *M*. *gordonae*. Polymerase chain reaction was performed to amplify mycobacterial DNA, and the amplified DNA was sequenced by using 16S rDNA sequencing as previously described (*3*). By using a distance score of <1% from the sequencing library entry to identify the species, the isolate obtained from the skin lesions and the hot tub water was identified as *M*. *intermedium*.

## Conclusions

This is the first clearly documented case of granulomatous dermatitis caused by *M*. *intermedium*, a novel, slow-growing mycobacterium originally isolated from the sputum of a patient with pulmonary disease (*4*) and recently described in an elderly man (*5*). *M*. *intermedium* isolated from our patient's hot tub was responsible for a chronic granulomatous dermatitis, which appeared to be refractory to appropriate antimicrobial therapy because of repeated exposure to contaminated water. The nodular eruption resolved only when use of the hot tub was discontinued. The distribution of the skin lesions on his back corresponded to the position of the high-pressure water jets.

Mycobacteria are commonly recovered from various environmental and potable water sources. Covert et al. (*1*) isolated nontuberculous mycobacteria from 38% of sampled drinking water, and Collins et al. (*2*) found a wide variety of mycobacterial species (*M*. *kansasii*, *M*. *xenopi*, *M*. *avium*, *M*. *marinum*, *M*. *fortuitum*, *M*. *chelonei*, *M*. *gordonae*) from both domestic and environmental water sources. These organisms are inherently resistant to disinfectants such as chlorine, which contributes to their persistence, even in treated water (*6*).

Public and private hot tubs, whirlpools, and public spas are increasingly popular in the United States. Public spas are periodically inspected to ensure that minimum hygienic standards for water safety are maintained. A recent report, summarizing the results of several such inspections, suggests widespread violations (*7*); >50% of these inspections indicated significant deficiencies in disinfection, pH control, and general maintenance. Private facilities such as home hot tubs and whirlpools are not subject to any surveillance or quality control.

Several clinical syndromes have been attributed to waterborne mycobacteria. Several investigators (*8**–**11*) have reported an association between hypersensitivity pneumonitis and spa-associated contamination with *M*. *avium* complex. Aubuchon et al. (*12*) described a patient with an amputation stump infection caused by *M*. *fortuitum* acquired from a home hot tub, and Lee et al. (*13*) reported a 24-year-old woman who acquired a soft tissue infection caused by *M*. *abscessus* from a public bath where she was employed. A recent report (*14*) described an outbreak of lower extremity furunculosis caused by *M*. *fortuitum* that affected >115 patrons of a nail salon; culture of the water from the whirlpool foot bath showed contamination with *M*. *fortuitum*.

Our case report highlights the paramount importance of medical history in the care of patients with enigmatic illnesses. The patient's rash failed to respond to seemingly appropriate therapy over a 2-year period because of constant reexposure to the contaminated water. Had a familial outbreak occurred, the diagnosis may have been more obvious. In this case, the patient was the only person using the hot tub, and a point source was not suspected. Only with repeated questioning was an association with the hot tub established. Clinicians should consider asking patients about hot tub, whirlpool, and spa exposure in the appropriate clinical context, such as cutaneous disease or pulmonary infiltrates for which no clear explanation exists.
